# Diaphragm and Lung Transplantation

**DOI:** 10.3389/ti.2024.12897

**Published:** 2024-06-24

**Authors:** Alessandro Palleschi, Giovanni Mattioni, Antonella LoMauro, Emilia Privitera, Valeria Musso, Letizia Morlacchi, Maurizio Vergari, Daniele Velardo, Giacomo Grasselli

**Affiliations:** ^1^ Thoracic Surgery and Lung Transplantation Unit, IRCCS Foundation Ca’ Granda Ospedale Maggiore Policlinico, Milan, Italy; ^2^ Department of Pathophysiology and Transplantation, University of Milan, Milan, Italy; ^3^ School of Thoracic Surgery, University of Milan, Milan, Italy; ^4^ Dipartimento di Elettronica, Informazione e Bioingegneria, Politecnico di Milano, Milan, Italy; ^5^ Department of Healthcare Professions, IRCCS Foundation Ca’ Granda Ospedale Maggiore Policlinico, Milan, Italy; ^6^ Pneumology Unit, IRCCS Foundation Ca’ Granda Ospedale Maggiore Policlinico, Milan, Italy; ^7^ Neuropathophysiology Unit, IRCCS Foundation Ca’ Granda Ospedale Maggiore Policlinico, Milan, Italy; ^8^ Neuromuscular and Rare Diseases Unit, IRCCS Foundation Ca’ Granda Ospedale Maggiore Policlinico, Milan, Italy; ^9^ Department of Anesthesia, Intensive Care and Emergencies, IRCCS Foundation Ca’ Granda Ospedale Maggiore Policlinico, Milan, Italy

**Keywords:** review, lung transplantation, diaphragm, diaphragm dysfunction, phrenic nerve

## Abstract

Mutual interactions between the diaphragm and lung transplantation (LTx) are known to exist. Before LTx, many factors can exert notable impact on the diaphragmatic function, such as the underlying respiratory disease, the comorbidities, and the chronic treatments of the patient. In the post-LTx setting, even the surgical procedure itself can cause a stressful trauma to the diaphragm, potentially leading to morphological and functional alterations. Conversely, the diaphragm can significantly influence various aspects of the LTx process, ranging from graft-to-chest cavity size matching to the long-term postoperative respiratory performance of the recipient. Despite this, there are still no standard criteria for evaluating, defining, and managing diaphragmatic dysfunction in the context of LTx to date. This deficiency hampers the accurate assessment of those factors which affect the diaphragm and its reciprocal influence on LTx outcomes. The objective of this narrative review is to delve into the complex role the diaphragm plays in the different stages of LTx and into the modifications of this muscle following surgery.

## Introduction

Lung transplantation (LTx) is a well-established treatment for benign end-stage pulmonary diseases in selected patients. During the entire LTx process, numerous factors can interfere with the function and the morphology of the diaphragm, the main respiratory muscle. Starting from the preoperative phase, the diaphragm may be influenced by patient-specific and disease-related variables, such as the underlying respiratory disease, the comorbidities and the chronic treatments [1, 2]. These interactions persist throughout the postoperative period, beginning with the surgical intervention, which itself can represent a stressful trauma for the diaphragm. Conversely, the diaphragm can influence the different phases of LTx. The presence of a diaphragmatic dysfunction before LTx, may require a different patient management. During surgery, the volume of the recipient’s chest cavity can depend on the diaphragmatic morphology leading to a size-matching issue. In the post-LTx setting, diaphragmatic dysfunction may hamper respiratory weaning, and impact on the long-term respiratory function of the patient or on sleep-related disorders [3–6]. Despite all these relevant dynamics, little is known on the real effects of diaphragmatic function abnormalities on LTx patients and *vice versa*. Notably, a standardized framework for the evaluation, definition, and management of diaphragmatic dysfunction within the context of LTx has yet to be established. This deficiency hampers the accurate assessment of those factors which affect the diaphragm and its reciprocal influence on LTx outcomes. The primary objective of this narrative review is to delve into the intricate role the diaphragm plays in the different stages of LTx and into the modifications of this muscle following surgery, along with an overview of fundamental aspects of diaphragmatic function.

## The Diaphragm

The diaphragm is the main respiratory muscle. Its contraction, along with that of the other respiratory muscles, generates sub-atmospheric pressure in the pleural cavity, creating a pressure gradient for air entry into the lungs. At rest, expiration is a passive process relying on the elastic recoil of the inflated lungs, whereas during exercise, expiration becomes active [7].

The diaphragm is a dome-shaped muscle, composed of vertical muscular fibres originating from a central tendon. It serves as the anatomical division between the thoracic and the abdominal cavity and it is innervated by the phrenic nerve. The diaphragm accounts for about 70% of the inspired air volume during quiet breathing: the muscle contracts with a piston-like movement causing a flattening of the dome and a decrease in the intra-thoracic pressure, thus allowing lung inflation. The pressure is generated against resistive and elastic loads that depend on airway resistance and chest wall compliance [7–9].

The function of the diaphragm is affected by many pathological mechanisms and physiological variables (e.g., level of consciousness, posture, lung expansion, lung compliance). The diaphragm’s inability to maintain adequate ventilation can be caused by interference with innervation, contraction, or mechanical coupling to the chest wall [10, 11].

### Diaphragmatic Dysfunction: Definition and Presentation

A diaphragmatic dysfunction can be defined as a loss of the function of the diaphragm, namely, respiration. It can be uni- or bi-lateral, transient or permanent, partial (weakness) or complete (paralysis), and its clinical significance can be variable. The clinical spectrum of diaphragmatic dysfunction is diverse, spanning from asymptomatic individuals to those experiencing severe respiratory failure. This can be secondary to a wide variety of factors related both to the characteristics of the dysfunction (e.g., bilateral vs unilateral) and the patient (e.g., obesity, lung disease) [12, 13]. In bilateral dysfunction, symptoms are more commonly present and intense. Conversely, a unilateral diaphragm dysfunction may often result asymptomatic. When present, symptoms may include orthopnoea and dyspnoea during exertion. Additionally, a diaphragmatic dysfunction can be linked to sleep-related breathing disorders, particularly in obese individuals. In cases of more pronounced diaphragm paresis, it can lead to snoring, breath cessation, and daytime sleepiness [14]. In the LTx setting, the respiratory function of the patient is already compromised due to the underlying respiratory disease, and this may interfere with a clear understanding of the role of diaphragmatic function abnormalities.

### Techniques for Assessing Diaphragmatic Function

The diaphragmatic function can be evaluated through specialized tests, although not all are routinely available in clinical practice. However, several common diagnostic tools can also be employed to suspect or explore a potential diaphragm dysfunction. Some of these tests are based on evidence that are not specifically conceived on LTx patients. The presence of an underlying respiratory disease should always be kept in mind because it could overshadow the interpretation of diaphragmatic function.

Standard respiratory function tests are routinely available and easily accessible, thus even if not specific, in case of abnormalities (e.g., reduced forced vital capacity - FVC) they may rise the suspicion of diaphragmatic dysfunction [15, 16].

Supine respiratory function tests are the only specific pulmonary function tests available for diaphragmatic evaluation [16, 17]. A supine reduction of more than 30% or 15% of FVC is consistent with bilateral or unilateral diaphragmatic weakness, respectively.

The polysomnography is not specific for the diaphragm; however, it has been shown that unilateral diaphragmatic dysfunction has been linked to a higher prevalence of OSAS compared to healthy subjects [14, 18–22]. Thus, in patients suffering from diaphragmatic dysfunction, it may reveal a diagnosis of OSAS. On the contrary, in a LTx patient with a recently diagnosed OSAS, further testing for diaphragmatic dysfunction may be appropriate.

Even if diaphragmatic dysfunction may relate with exertional dyspnoea, exercise testing may not be specific for its diagnosis. Peripheral muscle weakness might determine a reduction in maximum oxygen consumption, overshadowing the detrimental effect of diaphragmatic weakness, and the inspiratory reserve is predominantly impacted by the thoracic respiratory muscles rather than the diaphragm [16, 23].

Pressure measurements are not always available in routine clinical practice but are specific. These tests measure the trans-diaphragmatic pressure (Pdi) as the difference between oesophageal and gastric pressures [16]. It is achieved through insertion of two balloon-tipped catheters through the nasal passage. Pdi can be measured during voluntary respiratory manoeuvres (e.g., sniff) or by inducing muscle contraction through electrical or magnetic phrenic nerve stimulation (TwPdi). The volitional nature of this test renders its reliability contingent upon patient effort and motivation.

The maximal inspiratory and expiratory pressures (MIP, MEP) involve assessing respiratory pressure during maximal efforts against a closed mouthpiece and indicate global respiratory muscle strength, thus they are not specific [15]. However, when MIP or SNIP are less than 60% or 30% of predicted values, unilateral or bilateral diaphragm paralysis, respectively, can be suspected.

Electroneuromyography (EMG) is specific and records action potentials of diaphragmatic muscle cells contraction [15, 16]. It investigates the electrical activation capacity of the diaphragm by surface or trans-oesophageal electrodes. Surface diaphragmatic EMG has certain limitations, including electrode placement accuracy, signal attenuation through interposing tissues, and potential crosstalk from adjacent muscles. Trans-diaphragmatic EMG, performed via a gastroesophageal catheter with an array of wire coils, offers a more precise assessment of diaphragmatic electrical activity. Both surface and trans-diaphragmatic EMG can be performed during volitional (i.e.,: sniff, maximal contraction) or non-volitional (i.e.,: transcutaneous electrical or magnetic nerve stimulation) tests. Possible abnormalities in diaphragmatic dysfunction may be a reduced motor output, an abnormal neuromechanical coupling during loaded breathing or an increased level of assistance during mechanical ventilation.

Optoelectronic plethysmography and respiratory inductance plethysmography are not routinely available diagnostic tools, and they are not necessarily employed to specifically detected a diaphragmatic dysfunction. These techniques involve the analysis of thoracic and abdominal surface motion [24, 25]. In case of bilateral diaphragm paralysis, an asynchronous motion (e.g., paradoxical breathing during inspiration) can be detected.

The chest X-Ray is one of the most employed tests to initially explore a suspected diaphragm dysfunction [26, 27]. In asymptomatic patient, it could commonly lead to an accidental diagnosis. It allows assessment of the shape and the position of the diaphragm. Several static parameters have been proposed to standardize the diaphragm evaluation. Most commonly, a right-sided dysfunction is defined when the hemidiaphragm is > 2–4 cm higher than the left side, whereas on the left side, the hemidiaphragm is at the same height or more than the right side. Another interesting measurement is the diaphragmatic height index (calculated as the ratio of the distance between the apexes of the two hemidiaphragms and the height of T10 vertebra) which may predict diaphragm paralysis effectively, demonstrating high sensitivity (>90%) and specificity (>85%) [28]. However, bilateral dysfunction is more complex to evaluate with a static chest X-ray.

Magnetic resonance imaging (MRI) is not commonly employed to assess diaphragmatic dysfunction in LTx. Static MRI studies provide data on muscle size and structure, which could reveal specific features of rare diseases [29]. It can also be employed for dynamic imaging of the thorax and diaphragm using non-contrast breath-hold sequences and free-breathing diffusion-weighted imaging. As an example, a relationship between craniocaudal diaphragmatic excursion, diaphragm fatty infiltration, pulmonary function tests, and abdominal volumes (as an index of diaphragm activity) was demonstrated in patients with Duchenne Muscular Dystrophy.

Ultrasonography is a cheap and accessible test that allows the analysis of diaphragm dome excursion, thickness (Tdi), and thickening fraction (TFdi) [4, 30]. The latter one is defined as the difference between end-inspiratory and end-expiratory thickness divided by end-expiratory thickness, expressed in percentage. Measurements can be made both in dynamic M-mode and B-mode. It should be noted that healthy individuals may frequently exhibit TFdi values exceeding 100%. Overall, a reduced thickening fraction to less than 20%–29% is considered significant for diaphragmatic paralysis.

Finally, fluoroscopy is considered the gold standard for diaphragmatic dysfunction diagnosis. It can be employed during sniff manoeuvres to assess diaphragm dysfunction [31]. A comparison between upright and supine fluoroscopy can also be useful. Findings of diaphragmatic dysfunction include a reduced or absent diaphragm excursion and a paradoxical motion (e.g., one hemidiaphragm ascending while the other descends).

The list of available diagnostic tests along with their interpretation is reported in Table 1.

**TABLE 1:** Available diagnostic tests to assess a diaphragmatic dysfunction.

Test	Details	Findings/interpretation
Standard respiratory function tests (non-specific) [15, 16]	Routinely available and easily accessible. Even if not specific, in case of abnormalities they may rise the suspicion of diaphragmatic dysfunction	• Reduced VC • Normal or increased RV • Decreased TLC • Decreased FVC • Decreased maximum expiratory flow • Normal DLCO
Supine respiratory function tests (specific) [16, 17, 32]	Only specific pulmonary function test available for diaphragmatic evaluation	Bilateral diaphragmatic weakness • Seated FVC <50% of predicted • Supine decrease of FVC >30% Unilateral diaphragmatic weakness • Seated FVC <80% of predicted • Supine decrease of VC higher than 15%
Polysomnography (non-specific) [14, 18–21]	Even if not specific, it has been shown that unilateral diaphragmatic dysfunction has been linked to a higher prevalence of OSAS compared to healthy subjects	• Sleep hypopnea in moderate to severe diaphragmatic weakness • Increased severity of sleep disorder breathing (particularly during the REM phase, where the diaphragm is the primary inspiratory muscle) • Reduced responsiveness to CPAP • Higher incidence of necessitating BPAP.
Exercise testing (non-specific) [16, 23]	Potential overshadowing from peripheral muscle weakness	Reduced exercise tolerance
Pressure measurements (specific) [16]	Trans-diaphragmatic pressure (Pdi) is the difference between esophageal and gastric pressures, measured through the insertion of two balloon-tipped catheters through the nasal passage. There are voluntary Pdi (e.g., sniff) or induced Pdi through nerve stimulation (TwPdi) Voluntary Pdi limit is its volitional nature	Unilateral diaphragm paralysis • TwPdi <10 cmH₂O with unilateral phrenic nerve stimulation Bilateral diaphragm paralysis • TwPdi <20 cmH₂O with bilateral phrenic nerve stimulation Clinically significant inspiratory muscle weakness can be excluded when sniff-Pdi or Pdimax exceed • 80 cmH2O for men • 70 cmH2O for women
Maximal inspiratory and expiratory pressures (MIP, MEP) (non-specific) [15]	It assesses respiratory pressure during maximal efforts against a closed mouthpiece and indicate global respiratory muscle strength	Unilateral diaphragm paralysis • MIP or SNIP <60% predicted values Bilateral diaphragm paralysis • MIP or SNIP <30% predicted values Clinically significant inspiratory muscle weakness can be excluded when SNIP exceed • 70 cmH2O for men • 60 cmH2O for women Or MIP exceed • 80 cmH2O for men • 70 cmH2O for women
Electroneuromyography (EMG) (specific) [15, 16]	EMG records action potentials of diaphragmatic muscle cells contraction by surface or trans-esophageal electrodes. Both EMG can be performed during volitional (i.e.,: sniff, maximal contraction) or non-volitional (i.e.,: transcutaneous electrical or magnetic nerve stimulation) tests	• Reduced motor output • Abnormal neuromechanical coupling during loaded breathing • Reduced efficacy of contraction (when associated to ventilation measurements) In the ICU • Increased level of assistance during mechanical ventilation • Increased effort to breathe
Optoelectronic plethysmography and respiratory inductance plethysmography (non-specific) [24, 25]	Not routinely available and not necessarily employed to specifically detected a diaphragmatic dysfunction. They analyse thoracic and abdominal surface motion	Bilateral diaphragm paralysis • Asynchronous motions (e.g., paradoxical breathing during inspiration) Unilateral diaphragm paralysis • Chest asymmetry
Chest X-Ray (specific) [26–28]	One of the most employed. Bilateral dysfunction is more complex to evaluate	• Elevated hemidiaphragm (frontal, end-inspiratory chest X-ray), in particular o on the right side, the hemidiaphragm is > 2–4 cm higher than the left side o on the left side, the hemidiaphragm is at the same height or more than the right side • Inspiratory-expiratory difference between the two hemidiaphragms
Magnetic resonance imaging (MRI) (non-specific) [29]	Not commonly employed. Both static and dynamic MRI can be performed	• Reduced craniocaudal excursion • Diaphragm fatty infiltration
Ultrasonography (specific) [4, 30]	Cheap and accessible. It shows diaphragm dome excursion, thickness (Tdi), and thickening fraction (TFdi). TFdi is the difference between end-inspiratory and end-expiratory thickness divided by end-expiratory thickness, expressed in percentage	• Reduced thickening fraction to less than 20%–29%
Fluoroscopy (specific) [31]	Gold standard. Upright and supine comparison can be useful	• Reduced or absent diaphragm excursion • Paradoxical motion (e.g., one hemidiaphragm ascending while the other descends)

VC, vital capacity; RV, residual volume; TLC, total lung capacity; FVC, forced vital capacity; DLCO, diffusing lung capacity of carbon monoxide; REM, rapid eye movement; CPAP, continuous positive airway pressure; BPAP, bi-level positive airway pressure; MIP, maximal inspiratory pressure; MEP, maximal expiratory pressure; SNIP, sniff nasal inspiratory pressure.

## The Diaphragm in the Lung Transplantation Process

The intricate relationship between LTx and the diaphragm extends across various stages of the transplantation process. This interplay can be simplified in two main phases: before and after LTx.

### The Diaphragm Waiting for Lung Transplantation

**FIGURE 1:**
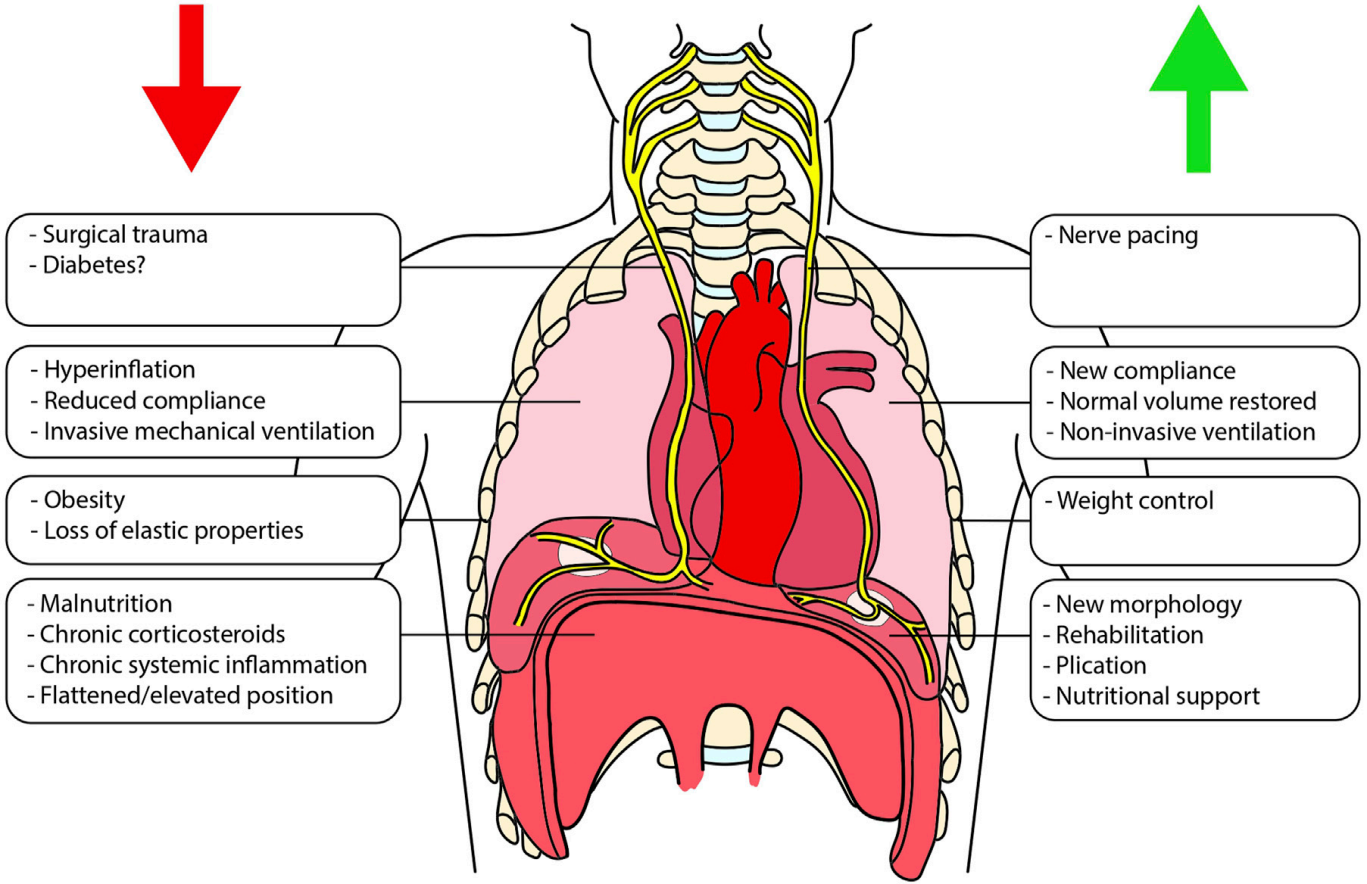
Schematic representation of the different factors that can impact the diaphragmatic function both before and after lung transplantation. On the left (red arrow) are shown factors that can negatively interfere with diaphragmatic function, whereas on the right (green arrow), are listed the promoting ones.

Several factors can influence diaphragmatic function in patients waiting for LTx (Figure 1) [10].

Patients suffering from obstructive respiratory disorders, such as chronic obstructive pulmonary disease (COPD) and cystic fibrosis, experience chronic overexertion of respiratory muscles due to the increased resistance necessary for adequate lung inflation [33, 34]. Furthermore, in the presence of hyperinflated lungs characterized by an increased functional residual capacity (FRC), the diaphragm is flattened and operates at suboptimal lengths, resulting in fatigue and dyspnoea during physical activity [35]. In addition, this may eventually lead to a paradoxical inward movement of the lower ribcage margin during inspiration, known as the “Hoover sign” [36]. Evidence indicate that COPD patients exhibit diminished voluntary and induced respiratory pressures, including MIP, Pdi and TwPdi [35]. Notably, MEP emerges as an independent risk factor for survival in COPD patients [37]. Studies have also revealed a correlation between abnormalities in diaphragmatic morphology, assessed via CT-scan, and the severity of COPD [38]. Chronic overexertion of the diaphragm is also found in restrictive respiratory disorders, such as idiopathic pulmonary fibrosis [39, 40]. In this case, the diaphragm may result elevated, due to reduced lung compliance. However, the overload following the increased stiffness of the lung may not only cause exhaustion, but also respiratory muscle training [40]. Some Authors speculate that the presence of chronic systemic inflammation may also play a role in respiratory muscles deterioration [40].

Diabetes is a well-known cause of neuropathic damage and can lead to a muscular deficit too [41]. Its prevalence is consistent even in the cohort of patients waiting for LTx, reaching up to 25% [42]. However, the relationship between diabetes and phrenic nerve or diaphragm dysfunction remains unclear [3].

Nutritional status plays a pivotal role both before and after LTx [43]. Obesity has been linked to increased post-LTx mortality [43, 44] and sparks debate about potential structural alterations in the human diaphragm [2, 45]. Obesity is also the most common restrictive disorder, exerting a higher body mass load both on the chest wall and on the abdomen, leading to an increased respiratory workload [10]. A correlation between malnutrition and diminished diaphragmatic strength in patients with cystic fibrosis was found [46], and some post-transplantation studies have reported reduced survival in malnourished individuals [47, 48]. Nevertheless, the relationship between nutrition and diaphragm function and structure remains a complex and incompletely understood area of study [47].

Both acute and chronic inflammation may affect the respiratory muscle function [49]. Some Authors speculate that in respiratory diseases with chronic systemic inflammation, such as idiopathic lung fibrosis, this mechanism may participate in muscle weakness [50]. Inflammation may also result from infections, especially in patients affected by cystic fibrosis [51]. Patients affected by severe respiratory diseases may also experience acute septic states. Although sepsis has been associated with temporary and reversible diaphragm dysfunction [52], more evidence is needed to fully grasp the correlation between acute inflammation and abnormal diaphragmatic function.

Chronic corticosteroid use-related myopathy is a known adverse effect and can affect function and volume of skeletal muscles [1, 53, 54]. Even if there are no studies specifically addressing the diaphragm in humans, particularly those waiting for LTx, a loss of respiratory strength has been described in chronic corticosteroid treatment. However, it is reasonable to postulate the presence of multiple confounding factors that warrant consideration (e.g., patients with worse respiratory function and overall health possibly receive higher corticosteroid doses compared to healthier counterparts).

Finally, another pre-LTx factor that may influence the diaphragm is the respiratory support. The impact of chronic non-invasive ventilation (NIV) on diaphragm function remains underexplored. While some Authors suggest potential improvements in diaphragmatic contraction with NIV, other studies report no discernible effects [55]. The impact of NIV on patient survival remains controversial. Nonetheless, NIV is a widely employed treatment for chronic respiratory failure and is accepted as a bridge to LTx [56]. Conversely, invasive mechanical ventilation has been associated with diaphragm atrophy, with links to prolonged mechanical ventilation, ICU admission, and complications during acute respiratory failure [57]. Currently, there is a lack of data to clarify the impact of extracorporeal membrane oxygenation (ECMO) on diaphragmatic function, in the setting of bridging to LTx [58, 59].

### The Interplay Between the Diaphragm and Lung Transplantation

LTx potentially triggers significant alterations in the diaphragm. Concurrently, the diaphragm influences the course of LTx (Figure 1). Herein, we provide an overview of the key aspects governing the reciprocal relationship between the two.

#### Diaphragm Activity, Outcomes, and Respiratory Function

Diaphragm contraction depends on various factors (e.g., neural function, diaphragmatic morphology and structure), although isolating their individual significance can be challenging. The incidence of a diaphragmatic dysfunction can vary across studies due to different identification methods and definitions [3, 27]. When referring to multiple parameters, such as respiratory function test, chest X-ray, ultrasound, opto-electronic plethysmography, and electromyography, in the early postoperative phase of LTx a diaphragm dysfunction may be systematically detected, although it might not necessarily be clinically significant [3]. This was shown in a cohort of 30 BTLx patients and the abnormalities persisted for 6 months, with full recovery at one-year post-transplantation [3]. Diaphragmatic dysfunction has been noted in terms of force, weakness, electrical activity, and kinematics. Despite that, an improvement in global spirometry and the six-minute walking test (6MWT) was reported. An incidence of 62% for diaphragmatic dysfunction was observed using ultrasound assessment, which decreased till 22% at 3 months from LTx, without impacting outcomes [60]. When concerning only chest X-ray findings, a unilateral elevated hemidiaphragm was detected in 23% of a cohort of 1,100 LTx patients in the early postoperative period (with a median of 21 days post-surgery) [27]. This abnormality reverted in 38% of cases, but in the remaining 62% a permanent elevation over time was seen, though no significant impact on outcomes (e.g., survival, chronic lung allograft dysfunction) was noted despite worse lung function tests. Additionally, a diaphragmatic elevation was present before LTx in nearly 3% of the study cohort, and this was a significant risk factor for presenting postoperative diaphragmatic elevation (*p* < 0.001), predominantly permanent (*p* < 0.001). More than half (58%) of these patients had pulmonary fibrosis as indication to transplantation. Interestingly, diaphragm elevation reverted after LTx in 45% of cases.

Huh et al. analysed the clinical relevance of a pre-LTx diaphragmatic dysfunction and its possible evolution after transplantation. Of 102 BLTx patients, 32% presented preoperative diaphragmatic dysfunction during ultrasound assessments [61]. After surgery, 12% and 3% of them showed a persistent (same side) and new (contralateral side) dysfunction, respectively. Moreover, nearly 55% recovered at 3 months from surgery, and an additional 30% within one-year. The presence of preoperative diaphragmatic dysfunction was found to be a negative prognostic factor. These patients experienced prolonged mechanical ventilation, extended ICU and hospital stays, and showed a significantly lower FVC. The difference in in-hospital mortality between patient with and without pre-LTx dysfunction was not statistically significant. However, the subgroup with the highest mortality was the one with preoperative diaphragmatic dysfunction that did not recover at 3 months.

Diaphragm dysfunction following BLTx, identified by ultrasound, has been associated with difficult weaning in the ICU setting [4]. In patients experiencing challenging weaning, nearly 78% exhibited diaphragmatic dysfunction. Neuro-ventilatory efficiency (NVE), defined as the ratio of tidal volume to peak electrical activity of the diaphragm, was also linked to difficult weaning in these patients. Longer durations of ventilation inversely correlated with both TFdi and NVE.

The improvement of the diaphragmatic function goes in parallel to the respiratory function gain following BLTx, as demonstrated in a cohort of patients with cystic fibrosis and bronchiectasis [62]. The study monitored pulmonary function tests, MIP, and surface diaphragmatic electromyogram, since the pre-LTx phase. It was shown that maximal contraction strength and diaphragmatic resistance increased, and this positive effect tended to stabilize after 6 months post-surgery. In the early phase, at one-month post-LTx, MIP was not significantly improved, but the time limit (i.e., the duration between contraction onset and exhaustion) value was already substantially increased compared to the preoperative period. This could be attributed to increased Vital Capacity (VC) along with reduced hypoxic drive, allowing for longer breath-holding periods. However, diminished MIP values at this time may also be linked to pain or other inhibitory pathways. Noticeable MIP improvements tend to be perceived at 6 months post-LTx. In these cases, the negative correlation between MIP and RV/TLC (total lung capacity) observed pre-transplantation subsequently disappears. Another study showed that 2 years post-LTx, diaphragmatic and abdominal muscles’ thickness and strength were comparable to healthy controls [63]. However, quadriceps strength and cross-section were decreased by nearly 30% in LTx patients, with cumulative corticosteroid dosage emerging as an independent predictor of quadriceps atrophy.

When concerning long follow-up, in a cohort of 15 patients after 5 years post-surgery, lower diaphragm thickening ratios (DTR) but normal TFdi at FRC were found, and the DTR was unrelated to 6MWT distance but strongly correlated with forced expiratory volume in one second (FEV1) [64]. Nonetheless, when compared to normative data, most LTx patients exhibited nearly normal DTR values, indicating preserved or regained diaphragm contractility post-LTx. The presence of diaphragmatic muscular abnormalities was hypothesized after excluding a neural dysfunction, achieved through further testing of the phrenic nerve response to stimulation. This revealed normal electrical activity in both groups, but lower TwPdi in LTx patients. Whether these abnormalities existed pre-LTx or developed post-LTx remains unclear [64].

#### Neural Function

Neural activity, encompassing both the phrenic nerve and the central neural drive, plays a pivotal role in diaphragm function. During LTx surgery, the phrenic nerve is exposed to potential damage and subsequent dysfunction, which can range from complete (paralysis) to partial (neurapraxia or moderate axonotmesis). The duration of such dysfunction varies, spanning from permanent to temporary. The definition of phrenic nerve injury and, consequently, diaphragmatic dysfunction significantly varies across studies [3, 65, 66]. The incidence of phrenic nerve injury after LTx can range between 3%–43%, with complete permanent paralysis being relatively rare. Partial dysfunction potentially results from surgical manipulation (primarily of the pericardium and mediastinum) or the use of cold solutions/ice during surgery [3, 66]. When measuring diaphragmatic compound muscle action potential area and phrenic nerve latency, this dysfunction might be constantly present after LTx, with a return to normality over several months, and may be sub-clinical (i.e., asymptomatic, or not visible at chest X-ray) [3]. When the phrenic nerve injury is defined as the presence of both ultrasound and neurophysiological abnormalities, this incidence can reach almost 43% in LTx patients [66]. Around 29% of phrenic nerves exposed to injury during surgery on the same side possibly sustain damage. Identified risk factors include right lung grafts and mediastinal adhesiolysis. LTx patients with phrenic nerve injury often experience longer ICU stays, increased reintubation rates, and more frequent use of NIV [66, 67]. Nevertheless, without a standardized definition and evaluation of phrenic nerve injury, determining its true impact on diaphragm function after LTx remains challenging. Moreover, the optimal management of this condition has yet to be defined. Even in the absence of detected diaphragmatic electrical activity, in the first 48 h post-LTx no respiratory impairment may be observed so far [68]. This finding emerged from a study involving the use of Neurally Adjusted Ventilatory Assist, which provides ventilatory assistance proportional to the diaphragm’s electrical activity. This activity was detectable in 63% of patients. Additionally, in two patients with long-term diaphragmatic dysfunction, normal electrical activity was recorded in the early postoperative period.

Another significant effect of LTx may relate to neural drive. In COPD patients undergoing LTx, a reduction in inspiratory effort sensation during ventilatory stress can lead to improved quality of life [69]. Compared to COPD patients, a decrease of the neural drive to the diaphragm with a normal endurance of inspiratory muscles was found after LTx. In single lung transplantation (SLTx) patients even the native side had a lower diaphragmatic neural activation. This suggests an involvement of the diaphragm at transplanted side to support the work of the other one.

Studying LTx patients, Kinnear et al. postulated the hypothesis that ventilatory compensation does not depend on vagal information from intrapulmonary or tracheal airway stretch receptors, but on diaphragmatic Golgi tendon organs [70]. In fact, with postural changes, the respiratory function tests revealed no differences between LTx patients and healthy controls. A tilt table test after the blockade of tracheal stretch receptors with aerosolized lidocaine have shown an immediate and unchanged ventilatory response. This hypothesis might be supported by the finding of unchanged respiratory pattern adaption to variations in ventilatory assistance and positive end-expiratory pressure (PEEP) in the early postoperative setting of LTx [68]. Notably, surgery determines interruption of vagal continuity at the bronchial anastomoses level, theoretically disabling the volume-feedback response.

#### Diaphragmatic Morphology

An interesting area of study concerns the modifications of diaphragmatic morphology following LTx and their consequent functional implications. In patient suffering from chronic lung hyperinflation, such as those with COPD, LTx reduces diaphragmatic flattening. This mitigation permits positional adjustments that bring mechanical advantages to ventilation, thereby achieving a respiratory gain [71]. One-month post-surgery, chest X-rays of LTx recipients can reveal extended diaphragm length compared to COPD patients. Additional findings may include higher sniff-Pdi values but similar TwPdi. Notably, the restoration of diaphragmatic morphology in emphysematous patients may need up to 2 years post-surgery [72]. Even in the presence of a positional recovery, the diaphragmatic surface may remain smaller on the graft side of patients receiving SLTx, when compared to the native side and to the ipsilateral side of healthy controls [72]. This phenomenon appears to be determined by a mediastinal displacement toward the transplanted lung. A similar finding was previously described by Groote et al. [73]. In addition, they demonstrated a bidirectional lateral movement of the mediastinum during respiration. Dynamic CT-scans of a single thoracic slice in both healthy controls and SLTx patients showcased mediastinal movement towards the native lung during inspiration and towards the graft during expiration. These movements seemed to be unaffected by changes in respiratory rate or position (supine or standing). However, it remains unclear whether these movements coincide with asymmetrical diaphragmatic motion.

Conversely, patients affected by restrictive disorders may tend to show a higher diaphragm. In a cohort of 37 SLTx patients, the pre- and post-LTx CT scans were compared to assess diaphragmatic changes. In restrictive disorders (i.e., fibrosis), while the native side had no modifications, diaphragmatic height significantly reduced on the graft side [74]. This may reflect an increased lung volume as well as an efficient diaphragmatic contraction with a more compliant lung, due to a previous chronic overload. In addition, the diaphragmatic thickness of the graft side also significantly increased in all patients. A negative association was found between diaphragmatic height and FVC and TLC, confirming the benefit of a diaphragmatic remodelling. However, diaphragmatic elevation in restrictive disorders may not always revert after LTx [27].

#### The Role of the Chest Wall

Following bilateral lung transplantation (BLTx) and heart-lung transplantation (HLTx), TLC tends to align with predicted values for recipients and does not correlate with pre-LTx values [75]. Nonetheless, despite TLC returning to normal values post-LTx, VC may decrease while FRC and residual volume (RV) increase. Normal FEV1/FVC ratio values might not be indicative of airway obstruction or muscle weakness but could signify irreversible alterations in the static elastic properties of the chest wall, possibly related to chronic lung hyperinflation. There is evidence concerning the role of the different surgical incisions (i.e., clamshell, thoracotomy, median sternotomy) on respiratory function and chest wall elasticity [76–78], however similar findings were not shown for the diaphragmatic function [3].

#### Diaphragm and Sleep-Disordered Breathing

Both LTx and diaphragmatic dysfunction are linked to sleep-disordered breathing [5, 6, 14, 19, 21, 22, 79]. In the setting of LTx, this sleep-related disorder can occur both before (18%–45% prevalence) and after (30%–64%) surgery, and patients without NIV or oxygen supplementation are at higher risk [5, 6, 22]. Interestingly, approximately 50% of pre-LTx sleep-disordered breathing cases may resolve after transplantation. The presence of this disorders was shown to not affect survival in LTx patients [5]. In non-LTx patients, diaphragmatic dysfunction was related to worse sleep-disordered breathing scenarios (e.g., increased respiratory disturbance index, lower oxygen saturation) and a different NIV management [21, 79]. However, literature lacks studies addressing the association between diaphragmatic dysfunction and sleep-disordered breathing in the LTx context. At present, only one case report exists, which showed that despite treatment and resolution of the diaphragmatic dysfunction in a LTx patient, the sleep disorder persisted [80]. Patients affected by sleep-disordered breathing usually require therapy (i.e., nocturnal NIV support) to alleviate symptoms, however the ideal management of a post-LTx sleep-related breathing disorder is yet to be defined [5, 6, 22]. Indeed, early diagnosis has been emphasized [6].

#### Postoperative Complications

Complications of LTx surgery may also directly involve the diaphragm. A spontaneous rupture of the diaphragm is a rare but possible complication of LTx [81, 82]. Literature cases have been described to appear in the early postoperative phase. Clinical manifestations include chest pain, subcutaneous emphysema, and dyspnoea. At chest X-ray abdominal visceral herniation in the thorax may be identified. The surgical repair usually consists of both a direct suture of the laceration or the positioning of a prosthesis (e.g., Goretex). A thinner diaphragm can be found intraoperatively. Some Authors suggested an association with lung emphysema.

### Potential Treatments

There is still no univocal evidence that a specific management of diaphragm function is effective in improving the outcomes after LTx. Many studies on this topic have been published and the interest continues to grow.

#### Diaphragm Plication

Lawrence et al. distinguished two indications for diaphragm plication: anatomical (i.e., size matching between recipient chest cavity and donor graft) and functional (i.e., clinically evident diaphragm dysfunction) [83]. They showed that most (78%) of the 38 diaphragmatic plication procedures were performed for anatomical reasons during LTx surgery, to increase recipient space and avoiding graft volume reduction. Almost 11% of patients receiving LTx during the study period underwent diaphragm plication, confirming the low frequency of this procedure. In the functional indication setting (22%), diaphragm plication was performed subsequently to LTx. Most were unilateral. Patients reported varying degrees of dyspnoea, orthopnoea, and persistent supplemental oxygen needs. Adhesions were almost constantly present, requiring an open surgical approach through a thoracotomy. Overall, unilateral right plication was the most common (57%), followed by bilateral plication (32%). A pre-LTx severe diaphragmatic dysfunction was detected on fluoroscopy only in 3% of patients. Interrupted sutures (62%) were more common than running sutures. Postoperative outcomes were satisfying. Only two (5%) asymptomatic patients had an incidental finding of liver laceration as a complication. In patients that received plication, the 6MWT distance at 1 year was not altered. When compared to patients without plication, FEV1 and FVC were consistently lower. Moreover, three-year survival and chronic lung allograft dysfunction-free survival were similar.

Other experiences of diaphragm plication in patients receiving LTx are limited mainly to case reports [84, 85].

This evidence confirms the extremely low frequence of diaphragmatic plication during LTx surgery for functional reasons. Future research could address whether it could be possible to identify ideal candidates that may benefit diaphragmatic plication at LTx time.

#### Non-Invasive Ventilation

Domiciliary NIV for post-LTx patients is uncommon; however, diaphragm palsy is one of the two main indications, as found in a retrospective study on 488 LTx over a 6.5-year period [86]. Five out of 20 (25%) patients requiring NIV had diaphragm dysfunction as an indication. Three LTx patients required NIV immediately after extubation while other two recipients started NIV within 1 month of being transplanted. All these patients had diaphragm palsies confirmed by ultrasound screenings. The incidence of diaphragm palsies in LTx patients ranged from 6.9% to 20%; however, only 1% of the patients required NIV for this indication. Further research is needed to further clarify the potential benefits of NIV in diaphragmatic dysfunction following LTx, along with its optimal introduction and discontinuation timing.

#### Physiotherapy and Nutritional Support

Respiratory physiotherapy is part of routine management in the early postoperative period after LTx and it is highly recommended [87, 88]. In the preoperative setting, respiratory rehabilitation has been shown to reduce dyspnoea and increase respiratory function parameters (e.g., 6MWT distance and DLCO) along with quality of life [89]. A trial showed that the addition of specific inspiratory muscle training (namely, the diaphragm) may empower the increase of walking distance, MIP and DLCO [90]. In the late post-LTx setting, respiratory rehabilitation is also linked to better respiratory performances [89]. It could be interesting to further investigate the optimal timing and methodology to rehabilitate the diaphragmatic activity in patients both waiting and that received LTx, especially when a clinically evident diaphragmatic dysfunction is present. In the late postoperative period, a comparison of the respiratory rehabilitation results between patients with and without dysfunction may also help determine the ideal candidates for this treatment.

Currently, there are no studies directly addressing the nutrition status effect on the diaphragm in the LTx setting. However, given the existence of a relationship between the nutritional status and the diaphragmatic performance [91], it could be useful to examinate the effect of nutrition correction in pre- and post-LTx patients.

#### Phrenic Nerve Pacing

Phrenic nerve pacing consists in direct electrical stimulation of the phrenic nerve through surgically implanted electrodes, to support a diaphragmatic dysfunction. In a feasibility trial from the Leuven group on three LTx patients, it was shown that intrathoracic intermittent pacing may help wean from mechanical ventilation and reduce the incidence of diaphragm dysfunction [92]. The electrodes were implanted at the time of LTx surgery and removed after up to 7 days. Electric stimulation managed to trigger ventilation and offer monitoring of changes of the diaphragm activity. In another study with a larger cohort, 11 patients received a temporary pacing system positioned at LTx surgery, whereas five patients underwent a laparoscopic positioning of a chronic pacing system, remotely after LTx [93]. In these patients, it was demonstrated that diaphragm stimulation helped both weaning from mechanical ventilation and recovery from phrenic nerve injury. In selected high risk LTx patients, this technology may effectively manage both temporary and chronic diaphragmatic dysfunction.

## Conclusion

A renewed interest has recently emerged in understanding the role of the diaphragm in the LTx setting. Nonetheless, in this context, the identification and definition of diaphragmatic dysfunction remain highly heterogeneous across the literature. Furthermore, the clinical significance and optimal management of these diaphragmatic abnormalities are still unclear. Future research should focus not only on gathering more evidence but also on enhancing standardization of methods for assessment and treatment.
